# Computed tomography-guided endoscopic needle knife therapy for ileal pouch sinus

**DOI:** 10.1093/gastro/gov026

**Published:** 2015-07-10

**Authors:** Custon T. Nyabanga, Joseph Veniero, Bo Shen

**Affiliations:** ^1^Cleveland Clinic Lerner College of Medicine of Case Western Reserve University, Cleveland, OH, USA;; ^2^Department of Diagnostic Radiology, Cleveland Clinic, Cleveland, OH, USA and; ^3^Center for Inflammatory Bowel Diseases, Digestive Disease Institute, Cleveland Clinic, Cleveland, OH, USA

**Keywords:** endoscopic needle knife sinusotomy, ileal pouch-anal anastomosis, anastomotic leak, presacral sinus, computed tomography

## Abstract

Ileal pouch-anal anastomosis surgery can be complicated by anastomotic leaks, leading to the formation of abscess and chronic sinus that have been routinely managed by a surgical approach. We developed the endoscopic needle knife sinusotomy (NKSi) technique, which has become a valid alternative. The basic principle of endoscopic NKSi is dissection and drainage of the sinus through its orifice internally into the lumen of pouch body. The success of NKSi requires an access to the sinus from the pouch side. One of the most challenging situations for NKSi is a closed orifice of the sinus, which leaves an isolated chronic abscess cavity. Here we report a case of complicated presacral sinus with a closed orifice that was not amenable to NKSi, necessitating a CT-guided guide wire placement and subsequent NKSi.

## Introduction

Anastomotic leaks after ileal pouch-anal anastomosis (IPAA) surgery for ulcerative colitis (UC) or familial adenomatous polyposis occur in up to 10% of patients [1,2]. These leaks often lead to a sinus tract or cavity, which are common causes for pouch failure. The pouch sinus has traditionally been treated with fecal diversion by ileostomy, incision and drainage (I&D), surgical unroofing and pouch revision [3]. Our group developed the curative endoscopic needle knife sinusotomy (NKSi) therapy that has become a routine procedure at our center [4]. However, there are cases with complex presacral sinus that have not been amenable to NKSi. Among these is the complete closure of the orifice of the sinus, which precludes the access with the needle knife. Here we describe successful treatment of an encased presacral sinus with NKSi after placement of a transcutaneous guide wire under computed tomography (CT) imaging guidance.

## Case presentation

**Figure 1.**
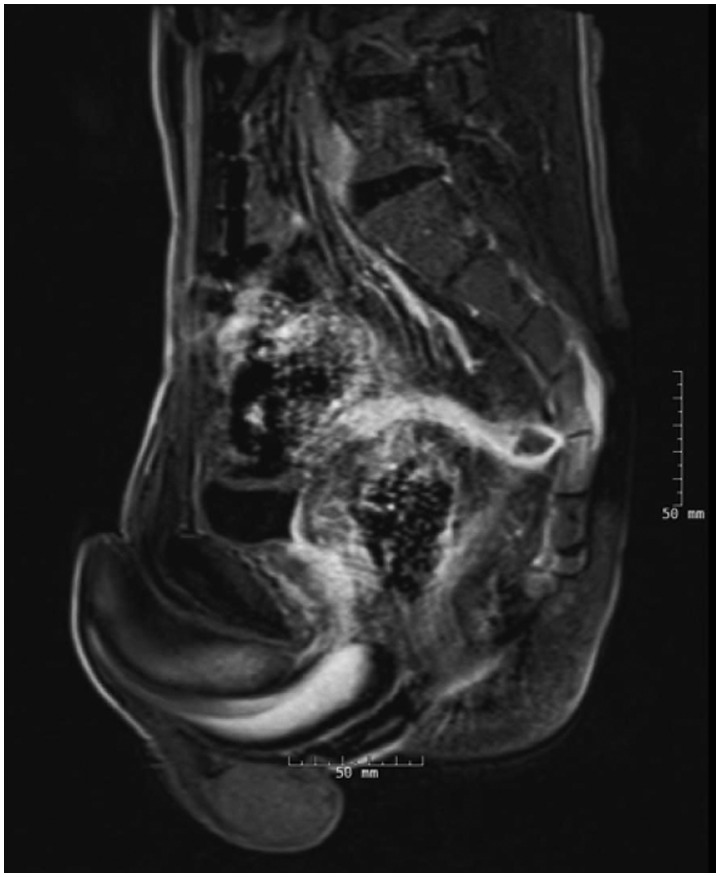
MRI images showing a fluid-containing tract extending from the presacral region at approximately the S2–S3 level. The tract extends anteriorly and encircles the mid portion of the pouch, well above the level of the anastomosis. No definite connection into the pouch is seen. The collection terminating in the presacral space measured 2.0 × 1.1 × 1.6 cm, with peripheral enhancement from active inflammation.

The patient was a 23-year-old male who presented to our clinic with abdominal pain, hematochezia and coccyx tenderness for five years after IPAA surgery. He was diagnosed as having UC in 2005, which became medically refractory, and he eventually underwent total proctocolectomy and two-stage IPAA in December 2008. Symptoms of abdominal cramping and increased bowel frequency developed immediately after ileostomy closure, which had initially responded to oral metronidazole and tetracycline therapy. At the time of presentation, he had been on long-term ciprofloxacin or metronidazole therapy for ‘pouchitis.’ Pelvic MRI six months prior showed a long sinus tract extending from the pouch anal anastomosis. A repeat MRI confirmed a fluid-containing tract extending from the presacral region anteriorly and encircling the midportion of the pouch above the level of the anastomosis (Figure 1). There was no definite connection to the pouch seen, and the sinus terminated in a small presacral collection measuring 2.0x1.1x1.6-cm^3^. Subsequently, a diagnostic pouchoscopy was performed and showed normal afferent limb, inlet, tip of J pouch body and cuff. The orifice of the presacral sinus was not identified on endoscopy, even by extensive probing with a soft-tip guide wire. In fact, the pouchoscopy showed a complete normal pouch anastomy and normal mucosa of afferent limb, pouch body, and anal transitional zone. A transanal endoscopic ultrasound was attempted and revealed a thick wall between the pouch body and sinus tract that precluded needle aspiration of the abscess.

**Figure 2.**
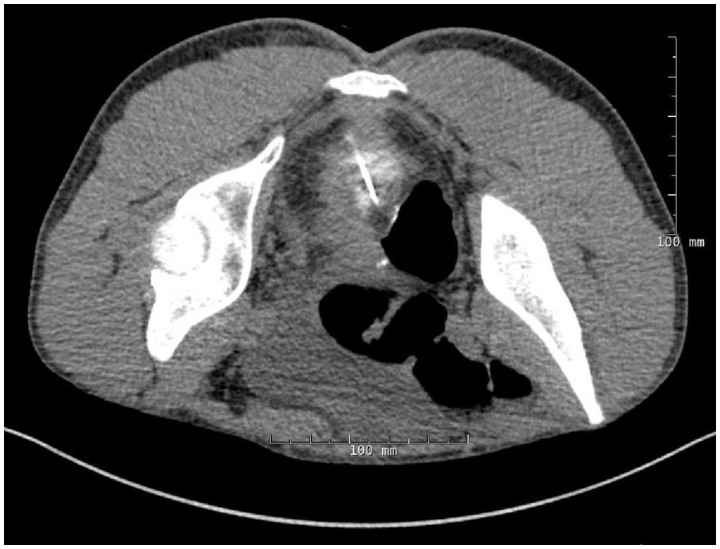
CT image showing thin guide-wire placement (Accura^™^ II breast localization needle) from buttock to posterior wall of the mid pouch.

Given the complexity of this sinus, we opted for CT-guided percutaneous placement of a guide wire to localize the sinus orifice. The sinus tract was localized using a needle from a 15-cm 20-gauge wire localization set and intermittent injection of dilute Omnipaque® 240 mg I/ml (1 ml diluted in 19 ml normal saline) contrast by the experienced radiologist (JV). With the tract identified, a small amount of gas was insufflated into the pouch via rectal catheter, and the needle was advanced into the pouch. The localization wire was advanced through the needle into the pouch, and the needle was removed. The localization wire was then secured to skin. CT imaging confirmed the placement of wire into the distal pouch body (Figure 2).

**Figure 3.**
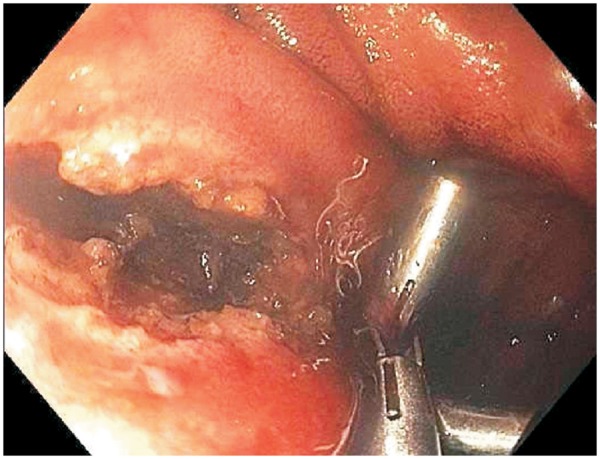
Endoscopic image showing a 4-cm sinusotomy draining into the posterior wall of the J pouch, with endoclips in place to keep the sinusotomy patent.

Subsequently, therapeutic pouchoscopy with NKSi was performed, with dissection of the sinus wall along the course of the wire. A 4-cm long sinusotomy was created, with dissected edges being stapled with endoclips to prevent reclosure of the orifice (Figure 3). The guide wire was removed after completion of NKSi. A follow-up pouchoscopy a month later showed an almost-healing sinusotomy with the endoclips in place. Further NKSi along with endoclips was performed to open up the sinus tract completely. The patient demonstrated a great improvement in symptoms and resolution of pain at the coccyx area.

## Discussion

Anastomotic leaks after IPAA surgery occur in up to 10% of patients, and medical management alone is often futile once an inflammatory tract or abscess forms [1,2,5]. Endoscopic methods for the management of presacral sinuses have gained favor, sparing most patients from the invasiveness of diversion ileostomy, redo pouch or reanastomosis surgery. Most of these cases have been managed with expectant management, sinus tract debridement, transanastomotic or percutaneous drainage or sinus unroofing [6]. Newer endoscopic techniques are increasingly being employed, including transanal unroofing, Endo-sponge® vacuum-assisted drainage, occlusive therapy with fibrin glue and needle-knife therapies, due to their lower invasiveness, shorter recovery time and low risk for complications. Some sinuses may heal spontaneously, but more than 50% persist, requiring more aggressive treatments [7–10]. Here we described a patient who developed a presacral sinus secondary to an anastomotic leak. Endoscopic NKSi alone was not successful due to the loss of access to the sinus. A multidisciplinary approach was undertaken. A CT-guided wire was placed to create an access for successful endoscopic NKSi. This patient would have otherwise been treated with surgical interventions.

The approach described here adds to the arsenal of endoscopic management techniques for presacral sinuses that often develop after IPAA surgery. This spares patients from the invasiveness and high risk of morbidity associated with the conventional surgical interventions. To our knowledge, this is the first case of successful CT-guided localization of a presacral sinus with a percutaneous guide wire to allow for endoscopic NKSi.

*Conflict of interest statement*: The authors declared no financial conflicts of interest. Dr. Bo Shen is supported by the Ed and Joey Story Endowed Chair.
